# Shortened Infant Telomere Length Is Associated with Attention Deficit/Hyperactivity Disorder Symptoms in Children at Age Two Years: A Birth Cohort Study

**DOI:** 10.3390/ijms23094601

**Published:** 2022-04-21

**Authors:** Cindy Pham, Regan Vryer, Martin O’Hely, Toby Mansell, David Burgner, Fiona Collier, Christos Symeonides, Mimi L. K. Tang, Peter Vuillermin, Lawrence Gray, Richard Saffery, Anne-Louise Ponsonby, on behalf of the Barwon Infant Study Investigator Group

**Affiliations:** 1 Murdoch Children’s Research Institute, Royal Children’s Hospital, Parkville, VIC 3052, Australia; cindy.pham@mcri.edu.au (C.P.); regan.vryer@mcri.edu.au (R.V.); martin.ohely@deakin.edu.au (M.O.); toby.mansell@mcri.edu.au (T.M.); david.burgner@mcri.edu.au (D.B.); christos.symeonides@mcri.edu.au (C.S.); mimi.tang@rch.org.au (M.L.K.T.); peter.vuillermin@deakin.edu.au (P.V.); richard.saffery@mcri.edu.au (R.S.); 2 Florey Institute of Neuroscience and Mental Health, University of Melbourne, Parkville, VIC 3052, Australia; 3 Melbourne School of Population and Global Health, University of Melbourne, Parkville, VIC 3052, Australia; 4 Child Health Research Unit, Barwon Health, Geelong, VIC 3220, Australia; fmcol@deakin.edu.au (F.C.); lekgrayresearch@gmail.com (L.G.); 5 Department of Paediatrics, University of Melbourne, Parkville, VIC 3052, Australia; 6 School of Medicine, Deakin University, Geelong, VIC 3220, Australia

**Keywords:** attention deficit/hyperactivity disorder, inattention/hyperactivity/impulsivity symptoms, telomere length, environmental factors, young children, epidemiology, infant secondhand smoke exposure

## Abstract

Environmental factors can accelerate telomere length (TL) attrition. Shortened TL is linked to attention deficit/hyperactivity disorder (ADHD) symptoms in school-aged children. The onset of ADHD occurs as early as preschool-age, but the TL-ADHD association in younger children is unknown. We investigated associations between infant TL and ADHD symptoms in children and assessed environmental factors as potential confounders and/or mediators of this association. Relative TL was measured by quantitative polymerase chain reaction in cord and 12-month blood in the birth cohort study, the Barwon Infant Study. Early life environmental factors collected antenatally to two years were used to measure confounding. ADHD symptoms at age two years were evaluated by the Child Behavior Checklist Attention Problems (AP) and the Attention Deficit/Hyperactivity Problems (ADHP). Associations between early life environmental factors on TL or ADHD symptoms were assessed using multivariable regression models adjusted for relevant factors. Telomere length at 12 months (TL12), but not at birth, was inversely associated with AP (*β* = −0.56; 95% CI (−1.13, 0.006); *p* = 0.05) and ADHP (*β* = −0.66; 95% CI (−1.11, −0.21); *p* = 0.004). Infant secondhand smoke exposure at one month was independently associated with shorter TL12 and also higher ADHD symptoms. Further work is needed to elucidate the mechanisms that influence TL attrition and early neurodevelopment.

## 1. Introduction

Telomeres are non-coding, repetitive nucleotide sequence structures located at the terminal ends of eukaryotic chromosomes and are essential for genomic stability [1]. Telomerase, a ribonucleoprotein complex responsible for progressive telomere maintenance, is expressed in most fetal cells, and its activity rapidly declines after birth [2,3]. Telomeres shorten after each DNA replication cycle, and once they reach a critical threshold, cellular senescence and apoptosis are initiated [4,5]. Telomere length (TL) measured at any time point in life is a biomarker of biological aging, reflecting the TL change and rate of shortening (‘attrition’) from birth [6,7].

Several environmental and genetic factors are known to influence TL shortening. In adults, this includes social adversity, smoking and secondhand smoke (SHS) exposure, chronic stress, and alcohol consumption [8,9,10,11,12]. Mechanistically, oxidative stress and inflammation appear to accelerate TL shortening [9,13,14]. The role of TL attrition in neurological diseases, including attention deficit/hyperactivity disorder (ADHD) [15] and Alzheimer’s disease [16,17], has been established in adults [18]. However, to date, few studies have investigated associations between TL and childhood neurodevelopmental disorders, with only one study examining the cases of ADHD [19], autism spectrum disorder (ASD) [20], and oppositional defiant behaviors [21]. One recent study reported on the association between TL and ADHD in adulthood and examined ADHD symptoms that persisted from childhood [15].

ADHD is one of the most common neurodevelopmental disorders in childhood. The diagnosis is based on the persistent patterns of ADHD symptoms mainly characterized by two behavioral traits: inattention and/or hyperactivity-impulsivity [22] impacting functioning and development. Globally, community prevalence is estimated to be between 2% and 7% among children under the age of 18 years [23,24]. ADHD is a major health condition, with onset as early as preschool years, that may persist into adulthood with a cumulative burden on the individual throughout the lifespan. These include social skills problems, mental health issues, academic challenges, and substance abuse [25,26,27,28]. However, an additional 5% of children presenting with substantial ADHD symptoms do not satisfy the full diagnostic criteria for an ADHD diagnosis [29]. Thus, this increases the greater risk for untreated ADHD in children, which may have significant negative impacts later in life. Previously, it was speculated that ADHD symptoms in children generally decline in adulthood [30,31]. However, epidemiological studies have reported that 30–60% of individuals that presented with substantial ADHD symptoms as children continued to have symptoms into adulthood [31,32,33]. Long-term negative impacts on these children later in life include lower education, comorbidity with other mental health disorders such as depression and anxiety, and work impairment [33]. 

Etiologically, ADHD is suggested to be a heterogeneous disorder due to both genetic and environmental factors. Despite known heritability among family studies, few candidate genes have been shown to contribute to the ADHD phenotypic variance [19,34]. Environmental factors, such as maternal lifestyle (e.g., tobacco smoking, alcohol, and drug use in pregnancy) [35,36,37,38] and perinatal factors, such as low birth weight, preterm birth, and birth complications (e.g., fetal distress) [39,40,41,42] may influence the pathogenesis of ADHD, particularly in early development [36]. We hypothesized that shorter TL in the first year of life may predict ADHD symptoms in children at age two years. In this study, we primarily investigated the association between cord and whole blood TL in the first year of life and ADHD symptoms in children at age two years. We then assessed the extent to which early life environmental factors and ADHD symptoms could be mediated through factors associated with TL shortening.

## 2. Results

### 2.1. Participant Characteristics

Characteristics of the Barwon Infant Study (BIS) participants are presented in Table 1 for the children assessed for ADHD symptoms in this study (n = 676). Infants were 53% male and 95% were full-term. On average, mothers were 32.1 years, and fathers were 34.0 years at the child’s birth; and for most families, both parents completed secondary school or the equivalent. Telomere length measurements were available at birth (TL0) for 64% (518/807) and at 12 months (TL12) for 76% of the children (477/629) with relevant behavioral assessments. 

**Figure 1:**
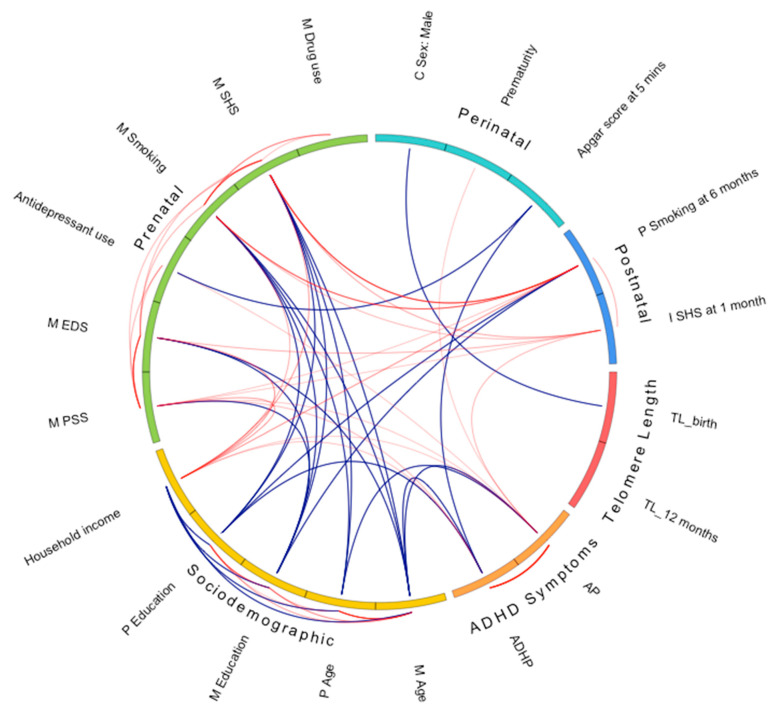
Pearson correlogram of all key early life environmental factors, telomere length in infancy, and attention-deficit/hyperactivity disorder symptoms at age two years. Key early life factors are represented as sociodemographic, prenatal, perinatal, and postnatal domains. Telomere length in infancy is indicated as the ‘Telomere Length’ domain. Attention-deficit/hyperactivity disorder symptoms are indicated as the ‘ADHD Symptoms’ domain. Intra-domain correlations are indicated by the lines outside of the circle. Inter-domain correlations are indicated by the lines inside the circle. For both intra- and inter-domains, the Pearson correlation limits are ≤0.10 and >0.10. Bolder lines indicate stronger correlations, while fainter line indicate weaker correlations. Positive correlations are indicated as red lines. Negative correlations are indicated as blue lines. ADHP— attention-deficit/hyperactivity problems; AP—attention problems; C—child; EDS—Edinburgh Depression Score; I—infant; M—maternal; P—paternal; PSS—Perceived Stress Score; SHS–secondhand smoke; TL–telomere length.

The Pearson correlations of all key early life environmental factors and TL in infancy, and ADHD symptoms at age two years, are shown in Figure 1. There was a strong correlation between primary outcome Child Behavior Checklist (CBCL) Attention Problems (AP) and secondary outcome Attention Deficit/Hyperactivity Problems (ADHP) (*r* = 0.85; *p* < 0.0001). Intra-domain correlations demonstrated that most of the sociodemographic factors were weakly to moderately correlated with each other (all, *p* < 0.001). Similarly, some of the prenatal factors were moderately correlated (Perceived Stress Score (PSS) and Edinburgh Depression Scale (EDS), *r* = 0.55, *p* < 0.0001; tobacco smoking and SHS exposure, *r* = 0.42, *p* < 0.0001) or weakly correlated, i.e., PSS/EDS with antidepressant use, tobacco smoking, and SHS exposure; *r* ranged from 0.09–0.13, all *p* < 0.001; recreational drug use was weakly correlated with maternal smoking and SHS exposure (*r* ranging from 0.11–0.26; *p* < 0.0001). None of the perinatal factors were correlated (*p* > 0.05). As for the postnatal factors, paternal smoking and infant SHS exposure at one month were weakly correlated (*r* = 0.15, *p* < 0.0001). Inter-domain correlations demonstrated that the majority of all early life environmental factors were correlated together. 

### 2.2. Telomere Length Measurements

**Figure 2:**
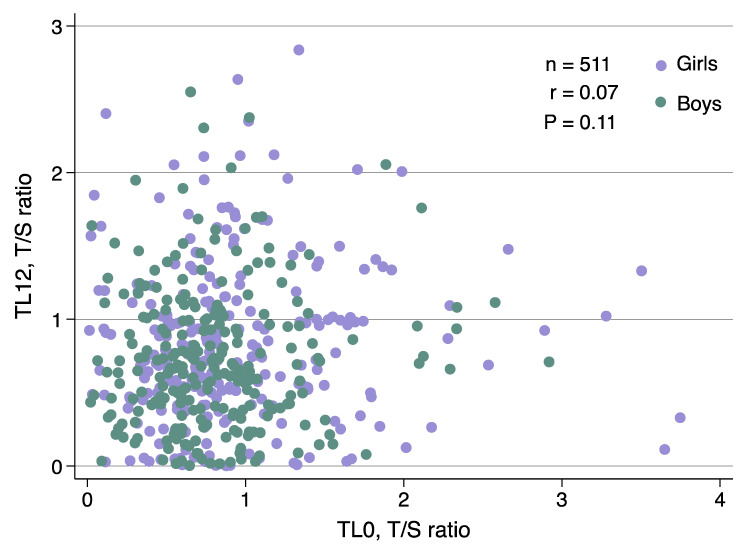
Telomere length distribution in infancy. Spearman correlation (r) between cord blood TL at birth and peripheral TL at 12 months. T/S ratio—telomeric genomic DNA/*β*-globin single-copy gene; TL0—telomere length at birth; TL12—telomere length at 12 months.

The mean intra-assay percent coefficient of variation (%CV) was 1.70% (standard deviation (SD) 2.20%; range 0.50% to 2.20%) for the quadruplicates, and the inter-assay %CV between plates was 3.60% (SD 1.82%; range 0.90% to 6.60%). The intra-class correlation coefficient (ICC) was calculated for measurements of the same type (TL or human beta-globin gene (*HBB*)), DNA controls (K562 cell line with ‘short’ TL, cord blood with ‘normal’ TL, and placenta with ‘long’ TL), and dilution concentrations (0.2, 1.0, 5.0, 10.0, 20.0 ng/μL) run in sets of quadruplicates. The intra-assay individual ICC was 0.99; 95% confidence interval (CI) (0.98, 1.00) and the inter-assay individual ICC was 0.96; 95% CI (0.91, 0.99) between 48 plates. TL0 (unit: relative amount of telomeric DNA (T) to a single copy gene (S, i.e., *HBB*); T/S ratio) ranged from 0.004 to 3.75 and the TL12 (T/S ratio) ranged from 0.002 to 2.84. TL0 was not correlated with TL12 (*r* = 0.07; *p* = 0.11; Figure 2).

### 2.3. Univariable Model with Infant Telomere Length and ADHD Symptoms at Age Two Years 

Estimates of the univariable model of the associations between TL0 and TL12 with ADHD symptoms at age two years are shown in Table 2. There was an inverse association between TL12 and both AP (regression coefficient (*β*) = −0.60; 95% CI (−1.14, −0.05); *p* = 0.03) and ADHP (*β* = −0.70; 95% CI (−1.12, −0.27); *p* = 0.001). Longitudinal birth and 12-month TL measures were included in the same model (referred to as the mutually adjusted model hereafter); the association with TL12 persisted for ADHP and attenuated for AP. There was less evidence of an association with TL0, so we focused on TL12 hereafter.

### 2.4. Early Life Factors Associated with TL12 and ADHD Symptoms at Age Two Years

Key sociodemographic and environmental factors that were independently associated with either TL12 or ADHD symptoms at age two are shown in Table 3. There was an inverse association between paternal education and TL12 (*β* = −0.08; 95% CI (−0.16, −0.001); *p* = 0.05), and ADHP (*β* = −0.08; 95% CI (−0.16, −0.001); *p* = 0.05). Greater infant SHS exposure at one month was the only environmental factor that was independently associated with shorter TL12 (*β* = −0.29; 95% CI (−0.53, −0.06); *p* = 0.02) and also increased ADHD outcomes: AP (*β* = 3.34; 95% CI (0.79, 5.90); *p* = 0.01) and ADHP (*β* = 2.27; 95% CI (0.43, 4.12); *p* = 0.02). For an extension of associations between early life factors with either TL12 or ADHD symptoms, see Supplementary Table S4. We then assessed whether the aforementioned factors (i.e., paternal education and infant SHS exposure at one month) were common causes, mediators, or determinants of the ADHD outcomes. There was weak evidence of a mediation effect by shorter TL12 for the effect of higher paternal education, and infant SHS exposure at one month on ADHD symptoms at age two years; thus, these were considered as potential confounders (see Supplementary Figure S2). 

Early life determinants of ADHD symptoms included maternal age, paternal age, household income, maternal prenatal perceived stress, maternal prenatal antidepressant use, maternal prenatal recreational drug use, prematurity, and paternal tobacco smoking at six months (Table 3). Using the change in estimates approach and given the strong correlation between maternal age and paternal age, paternal education, paternal smoking at six months and household income, only maternal age and household income were included in the final multivariable model. The multivariable model was adjusted for process factors to minimize measurement error of: the exposure (i.e., child’s sex, child’s age at the time of the blood collection, time from blood collection to storage, and cell type composition), and of the outcomes (i.e., child’s sex, child’s age at the time of behavioral assessment), the determinants of ADHD symptoms including maternal age, household income, prenatal perceived stress, prenatal antidepressant use, prenatal recreational drug use, prematurity, and Apgar score at five minutes, and also further adjusted for a potential confounder: infant SHS exposure at one month.

### 2.5. Multivariable Model with Telomere Length in Infancy and ADHD Symptoms at Age Two 

Multivariable models of the associations between TL0 and TL12 with ADHD symptoms at age two years are shown in Table 2. The inverse association persisted between TL12 and both AP (*β* = −0.69; 95% CI (−1.29, −0.10); *p* = 0.02), and ADHP (*β* = −0.92; 95% CI (−1.39, −0.44); *p* < 0.0001). In the mutually adjusted model with both time points in the same model, the inverse association increased in magnitude of effect between TL12 and both AP (*β* = −0.66; 95% CI (−1.32, −0.002); *p* = 0.05) and ADHP (*β* = −1.12; 95% CI (−1.68, −0.57); *p* < 0.0001). 

### 2.6. Additional Analyses 

Shorter TL12 was associated with increased ADHD symptoms within the borderline to clinical range in the multivariable model (AP: OR = 0.26; 95% CI (0.09, 0.73); *p* = 0.01 and ADHP: OR = 0.39; 95% CI (0.18, 0.84); *p* = 0.02) (Supplementary Table S1). TL in infancy was categorized by median, quintiles, and the composite of both time points (Supplementary Table S2). There was a dose-response association between composite TL at both time points and the ADHD symptom outcomes, as well as a negative association between both long compared with both short TL in infancy and increased ADHD symptoms. There was modest evidence of an association by median or by quintiles (Supplementary Table S2). Furthermore, we conducted restricted analyses, excluding (i) seven infants with TL0 and one infant with TL12 that were deemed outliers, and (ii) 43 infants (5% of the cohort) with cord blood samples with potential maternal contamination (Supplementary Table S4). Inverse probability weighting was used to account for initial non-participation and attrition in the regression. All findings were consistent with the main results. There was weak evidence detected for the effect modifier by child’s sex for the associations between TL at both time points and ADHD symptoms and with other CBCL outcomes, including Autism Spectrum Problems and Oppositional Defiant Problems at age two years (all *p* > 0.05).

## 3. Discussion

This longitudinal study prospectively investigated the association between two TL measures in infancy and ADHD symptoms in young children. Our findings indicate that shorter TL12 was associated with increased ADHD symptoms in children at age two years, whereas TL0 was not. This association persisted following further adjustments for potential confounding factors. In the mutual adjustment model where TL12 was additionally adjusted for the baseline TL0 time point, there was a stronger magnitude of effect with TL12 with ADHD symptoms at age two years. Together, these findings highlight that the sensitive window of neurodevelopment in the first year of life may be markedly influenced by the early life environment, which may also explain the differences in TL at birth and at 12 months. We also found that approximately half of the infants had longer TL at 12 months compared to the length at birth. This is consistent with a recent study that observed a similar pattern, with longer TL in children at four years compared to TL at birth [43], and this pattern has been implicated in adult TL studies [44]. There are several possible explanations for this, including (i) the use of different biospecimen sources (cord blood vs. venous peripheral blood) and matrices (serum vs. plasma) that may yield different telomeric DNA levels [45], (ii) TL measurement misclassification remains possible, despite the high-quality TL data with low CV and high ICCs, and (iii) the qPCR method may give rise to some measurement error, as compared to other methods such as the Southern blot method [46]. These intriguing findings in this first study highlight the need for future work in other birth cohort studies to validate our findings and to delineate underlying mechanisms. Infants in the first year of life are particularly susceptible to environmental insults that may potentially accelerate TL shortening in the early phase of development, compared to later stages in life [43]. An alternative explanation is that TL0 may be closely related to maternal levels (see Supplementary Table S4), and thus may be protected by the maternal environment [47]. Moreover, there is emerging evidence on the epigenetic-like traits of TL where there is both an effect of genetic variants on TL [48,49], but where they are also influenced by DNA methylation [50]. Both heritable and environmental factors in early life contribute to TL dynamics and potentially the programming of TL longevity [47]. Highly heritable TL is greatly linked to genetics and environmental factors such as those related to parental or transgenerational influences, including parental age [51]. Both maternal and paternal age were colinear and both were associated with TL12 in the direction that corroborates previous work, i.e., older parents had offspring with longer TL. It is possible that the role of parental age on TL has different biological explanations. Increased maternal age may be a marker of a slower rate of biological aging and longevity, and this potentially reflects possible genetic variants that may play a role in exceptional survival [52,53], whereas increased paternal age is associated with elongated sperm TL [54,55], both of which may correlate with longer TL in the offspring. Furthermore, when assessing other early life environmental factors that are associated with the TL-ADHD association, we found that increased infant SHS exposure at one month was independently associated with shortened infant TL and increased ADHD symptoms at age two years. There was modest evidence of effect modification by child’s sex on the TL-ADHD association. 

To date, few studies in the literature have reported on the association between TL and ADHD in both children [19] and young adults [15]. The first cross-sectional study to demonstrate a link between leukocyte TL and ADHD symptoms was in school-aged children aged 6–16 years (n = 61) [19]. This study reported that shorter TL was observed in children with hyperactivity-impulsivity dimensions of ADHD, rather than inattention dimensions [19]. It is important to note the study was primarily focused on investigating parental TL effects on child TL with ADHD symptoms; thus, the longitudinal relationship between TL and subsequent ADHD symptoms was not addressed. On the contrary, one study reported that longer TL in adulthood was associated with increased ADHD symptoms in young adults aged 18–38 years who previously had an early onset of hyperactivity-impulsivity symptoms in childhood [15]. However, the study did not investigate the contribution of multiple factors over the lifecourse to TL, therefore limiting the study’s ability to unravel causal inference. There are emerging epidemiological studies that have reported on the associations between early TL and other neurodevelopmental outcomes in children that further corroborate our findings [20,21,56,57]. A study that used the same instrument to measure child behaviors as the present study investigated the association between TL and CBCL-oppositional defiant problems in children aged three to five years (n = 108) [21]. They reported that children with oppositional defiant behaviors at ages three and four had shorter TL than children without these behaviors [21]. However, this study lacked a baseline measure of TL; therefore, they were unable to investigate potential reverse causality. In this study with longitudinal TL measures, children with longer TL at both time points were less likely to have ASD problems and oppositional defiant behaviors compared to children with shorter TL (CBCL-autism spectrum problems and -oppositional defiant problems, respectively). Consistently, shortened TL in cord blood has also been associated with increased emotional and behavioral problems in the offspring [57]. In a cross-sectional study, shorter TL was observed in ASD-diagnosed children as compared to children without autism (n = 110) [20]. One longitudinal study investigated the association between TL at birth and five years of age and a number of neurodevelopmental outcomes at several ages (ranging from nine months to five years) (n = 184) [56]. This study reported an inverse association between longer TL and better scores in the psychomotor developmental index at age 30 months and on the Woodcock–Johnson Test of Achievement: Letter-Word Identification at age five years [56]. Comparisons of the present study with previous work are hampered by differences in the methods by which both TL and neurodevelopment were measured. Nevertheless, the trajectory of these previous studies demonstrates a similar pattern to this study, i.e., shorter TL is associated with increased childhood neurodevelopmental problems. 

The ‘developmental origins of health and disease (DOHaD)’ theory proposes that adverse environmental influences during in utero and the first few years of life have long-term programming effects, and this may be particularly true for the developing brain [58,59,60]. Here, SHS also refers to environmental tobacco smoke and passive smoking. We found that increased infant SHS exposure at one month was independently associated with both shorter TL12 and increased ADHD symptoms at age two. A recent meta-analysis of 18 longitudinal studies demonstrated that smoking does not influence TL shortening in adults [61]. Notably, these studies in the meta-analysis reported on the longitudinal effect of TL over the course of adulthood; they did not characterize whether participants were smoking during pregnancy or not, and neither reported its effects on the offspring. However, given the moderate correlation between infant SHS and maternal prenatal smoking, we assessed whether the association persisted between infant SHS and ADHD symptoms after further adjustments for maternal perceived stress, maternal prenatal smoking, and maternal prenatal SHS exposure that were correlated together (Figure 1 and see footnote, Table 3). We were unable to demonstrate that the impact of infant SHS exposure on ADHD symptomatology was mediated by TL12. One previous study reported that prenatal SHS exposure was associated with shorter TL in newborns [62]. In the present study, there was modest evidence of this association. Notably, only 11% of mothers were smokers during pregnancy; thus, the increase in infant SHS exposure at one month may partly reflect an accumulation of both parents’ smoking behaviors. Exposure to in-home SHS frequency of more than one hour per day has previously been linked to a three-fold increase in the risk of ADHD in children [63]. Furthermore, parental smoking has been shown to increase the risk of childhood ADHD [64]. In adults, SHS exposure has also been implicated in the accelerated rate of TL attrition [10,12]. Our finding corroborates previous evidence regarding the role of SHS exposure in the development and severity of childhood ADHD [63,65,66,67,68,69]. This present study adds to mounting evidence that SHS exposure during sensitive developmental windows is a preventable risk factor for subsequent ADHD. Future larger studies exploring the role of early environmental factors, such as infant SHS exposure, are needed to validate the link between infant TL and childhood ADHD.

To advance understanding of the role of TL in health and disease risk, it is important to further explore underlying biological mechanisms that influence the rate of TL attrition. Both oxidative stress and inflammation have been implicated in this process [70,71,72], and both have been linked to adverse neurodevelopmental outcomes, including ADHD [73,74,75,76,77]. Additionally, these pathways have also been linked to environmental exposures, such as SHS in children [78,79]. There is now increasing evidence that TL may be a proximal biomarker of these pathways, warranting further investigation of the nexus between telomere biology and molecular mechanisms (i.e., oxidative stress and inflammation) in relation to childhood ADHD.

### Strengths and Limitations

This study is the first and largest of its kind to directly assess longitudinal measures of TL in infancy and subsequent ADHD symptoms in young children. The strengths of this study are the population-derived, prospective measures and the serial telomere measures in the first year of life. Furthermore, the depth of data assembled in the BIS enabled the investigation of a broad range of environmental factors. The CBCL-AP has good predictive validity of ADHD diagnosis [80] and has high specificity of 0.91 [81]. This bolsters confidence in using AP to assess early childhood ADHD symptoms in this study. Another major strength of the present study is the high-quality TL measurements; we provided two indicators of measurement reliability, i.e., low intra- and inter-assay CV of less than 5% and ICCs that ranged from 0.96–0.99 [82,83]. TL was quantified by the quantitative polymerase chain reaction (qPCR) method, which has been widely used and validated against Terminal Restriction Fragment assays [84]. The qPCR method requires smaller quantities of DNA and permits high-throughput testing; thus, this method is well-suited for larger longitudinal studies. 

There are several limitations that should be noted. In the moderately sized cohort, some of the key exposures were relatively rare. For example, less than 3% of infants were subjected to SHS exposure postnatally, which may have contributed to inadequate statistical power, particularly for mediation analysis, whereby a larger sample size would have greater power [85]. Using different biospecimen sources of DNA at birth (cord blood) and at 12 months (venous whole blood) may have possibly yielded differences in the content of telomeric sequences [45]; in this regard, low correlations between TL at birth and 12-month are observed [43,86]. However, we also adjusted for cell type composition to minimize systemic bias. Given that not all early life factors were independent, multiple comparisons adjustments were not applied to minimize the risk of false-positive errors [87,88]. While some children may exhibit early signs of ADHD, older children are more likely to receive an official diagnosis at a later age; thus, follow-up studies are required among school-aged children. The comparison between our findings and previous studies was limited due to differences in TL and neurodevelopmental measurement methods. However, we provide good evidence for early life TL attrition and increased ADHD symptoms in young children, highlighting the basis for further longitudinal studies. 

## 4. Methods

### 4.1. Study Design and Participants

The BIS is a population-derived birth cohort study (n = 1074 infants); the objectives and methodologies of the study have been previously described [89]. In brief, women were recruited using an unselected antenatal sampling frame from two hospitals in the Barwon region of Victoria, Australia, between June 2010 and June 2013. Infants were excluded from the study if they were: (a) delivered before 32 weeks of gestation; (b) had major congenital malformations or a genetically determined disease; or (c) developed a serious illness [89]. Questionnaires, clinical data (i.e., perinatal factors), and biological samples were collected at several time points: at birth, at four weeks, at three, six, nine, twelve and eighteen months, and at two and four years of age (for more details on the selection of participants in this study, see Supplementary Figure S1). Study approval was granted by the Barwon Health Human Research and Ethics Committee (HREC 10/24), and informed written consent was provided by all parents or guardians.

### 4.2. Blood Collection and Processing

Umbilical cord blood was collected by syringe and added to 10 mL of RPMI 1640 (Gibco, Life Technologies, Massachusetts, USA) containing preservative-free sodium heparin (final concentration 10 IU/mL) (Pfizer, NY, USA), while venous peripheral blood collected at 12 months of age was added to a 15 mL tube containing 100 μL preservative-free sodium heparin (final concentration 10 IU/mL) (Pfizer, NY, USA). Samples were processed within 18 h of collection and a small aliquot (150 μL) of whole blood was cryopreserved at –80 °C until DNA extraction. Potential maternal-contaminated cord blood samples were identified using DNA methylation profiling as previously described by Morin and Gatev [90]. 

### 4.3. Telomere Length Measurements

Genomic DNA was extracted from mononuclear cells isolated from cord blood and 12-month whole blood using the QIAamp DNA QIAcube HT Kit (QIAGEN, Hilden, Germany) and quantified using the Qubit^®^ dsDNA Broad-Range Assay Kit (Thermo Fisher Scientific, Wilmington, DE, USA) following the manufacturer’s instructions and stored at −80 °C. The quantification and purity of genomic DNA were determined by the Qubit 2.0 Fluorometer (Thermo Fisher Scientific, Wilmington, DE, USA) and Nanodrop 1000 Spectrophotometer (Nanodrop Technologies, WI, DE, USA). Relative TL was measured by the widely used qPCR method, originally described by Cawthon [84]. Each sample was comprised of 4 μL of diluted genomic DNA at 5 ng/μL, 5 μL of SYBR^®^ Green PCR Master Mix (Thermo Fisher Scientific, Wilmington, DE, USA), and 0.5 μL of each forward and reverse primer at 2 μM, with a final volume of 10 μL for each reaction. The telomeric DNA primer sequences: CGGTTTGTTTGGGTTTGGGTTTGGGTTTGGGTTTGGGTT (forward) and GGCTTGCCTTACCCTTACCCTTACCCTTACCCTTACCCT (reverse), and the single-copy nuclear *HBB* gene primers sequences for GCAGGAGCCAGGGCTGGGCATAAAAGTCA (forward) and GGGCCTCACCACCAACTTCATCCACGTTC (reverse) were used for amplification.

In brief, after randomization, samples were transferred using a Sequenom MassARRAY Matrix Liquid Handler (Agena Bioscience, San Diego, CA, USA) to minimize pipetting error variability and systemic bias. Telomere and *HBB* qPCR reactions were performed on the same plate. The final plate layout included participant genomic DNA, three sets of genomic DNA controls, and a no-template control containing RNase-free water for each run. All assays were performed in quadruplicates using the Lightcycler^®^ 480 Instrument II (Roche, Melbourne, Australia) (i.e., 30 participant samples were measured per 384-well plate). The cycling conditions were: pre-denaturation at 95 °C for 10 min, followed by 30 cycles of (i) 95 °C for 15 sec and (ii) 62 °C for 2 min. Standard curves were obtained by serially diluting genomic DNA controls (ranging from 0.1 ng to 20 ng/μL; K562, cord blood, and placenta for different lengths of TL) and then integrated into a composite standard curve to assess qPCR efficiency. 

The qPCR efficiency was defined by the gradient of each standard and incorporated to determine the T/S ratio. Relative TL was obtained by calculating the ratio, known as the T/S ratio, by comparing the relative amount of telomeric DNA (T) to a single copy gene (S, i.e., *HBB*) for each sample to a reference genomic DNA. Samples were omitted from the analysis if results were out of the acceptable range (>0.5 crossing point (Cp) from the median). Approximately 15 samples were removed from the analysis due to not meeting this threshold and were not re-assayed. A total of 48 assay plates were used for TL measurements. Samples matched from both birth and 12-month time points were sequentially measured on the same plate to minimize positional effects.

### 4.4. Behavioral Assessment

The CBCL preschool version for children aged 1.5–5 years (CBCL 1.5–5) [91] is a 99-item standardized parent-reported measure designed to record the behavioral and emotional problems of children. It is a widely used [80,81,92,93] and validated measure with strong psychometric properties including high test-retest reliability [91,94]. Previously, AP has been reported to have good predictive validity of ADHD diagnosis in a prospective study of three-year-old children with an area under the curve (AUC) of 0.87 and 0.80 for girls (n = 238) and boys (n = 276), respectively [80]. Another study of young children aged three to five years with disruptive behavior disorders demonstrated favorable CBCL diagnostic utility properties, for example, high specificity of 0.91, sensitivity of 0.71, and a positive predictive power of 0.88 [81]. The CBCL produces seven Syndrome Scale scores, including: Emotionally Reactive, Anxious/Depressed, Somatic Complaints, Withdrawn, Sleep Problems, Attention Problems (AP), and Aggressive Behavior. Additionally, five Diagnostic and Statistical Manual of Mental Disorders (DSM-5)-Orientated Scale scores were derived including: Attention Deficit/Hyperactive Problems (ADHP), Autism Spectrum Problems, Anxiety Problems, Depressive Problems, and Oppositional Defiant Problems. Lacalle et al [95] recommended that orientated scale scores should be assessed simultaneously with syndrome scale scores for additional clinical information. Thus, this paper primarily focuses on both the AP (primary outcome) and ADHP (secondary outcome) as ADHD symptoms, reported as sex and age-normalized T scores. The T scores are derived from raw scores by standardization to a mean of 50 and an SD of 10 [91]; the mean (SD) for AP was 51.9 (3.7) and for ADHD, it was 51.8 (3.6). The BIS participant’s parent/guardian completed a questionnaire assessment about their child’s behavior at age two years. Of the 837, 676 children (81%) that participated had completed the CBCL assessment at the two-year follow up.

### 4.5. Other Factors

Key factors included in the analyses were based on a priori knowledge of previously reported associations with childhood ADHD [36,37,56] and other behavioral assessment in this cohort [96,97,98,99]. Given the low knowledge environment with determinants of telomere length in infancy, modern data-adaptive approaches [100,101] were used to deepen the understanding of potential confounders (Table 3 and Supplementary Figure S2). For an extended list of the other considered factors in this study, see Supplementary Table S3.

Sociodemographic factors including parental age at birth, parental education, and household income, and prenatal factors including maternal perceived stress, maternal depression, maternal smoking, and maternal SHS exposure, were collected by baseline questionnaires at around 28 weeks of gestational age. In brief, hardcopy self-assessed questionnaires were developed to facilitate the pooling of data with other Australian birth cohort studies, and the validity of the questionnaire items used have been examined and established, as previously described elsewhere [89]. Household income from lowest to highest (per AUD 10,000) was recorded as the mean of the values during pregnancy and one year after birth. Maternal perceived stress was measured by PSS, recorded as the mean of the values during pregnancy and the first six months postnatal [102]. The PSS is a validated measure, with adequate psychometric properties, for the assessment of perceived stress in women during prenatal and postnatal periods [103]. Maternal depression was measured by the EDS in trimesters one and two and four weeks postnatally, with scores below 10 indicating low risk, between 10–12 indicating moderate risk, and greater than 12 indicating high risk [104]. The EDS is widely used and validated for screening depression [104,105,106] in new mothers, a cut-off value of 11 or more in the first trimester and 10 or more in trimesters two and three for depressive symptoms has been demonstrated to have adequate sensitivity, specificity, and positive predictive value [104]. Both maternal smoking and maternal SHS exposure during preconception or pregnancy were classified as Yes (if any) vs. No. Perinatal factors, such as child’s sex, prematurity, and Apgar score at five minutes, were clinically collected and validated with hospital records [89]. Postnatal factors, including parental tobacco smoking at six months and infant SHS exposure at one month, were collected with follow-up repeated questionnaires. Process factors related to the outcome (child’s age at the time of behavioral assessment) and those of the exposure (child’s age at the time of blood collection, time interval between blood collection, and storage and cell composition to CD8 T-cells) were also collected. 

### 4.6. Statistical Analysis

#### 4.6.1. Participant Characteristics

Participant characteristics are summarized using descriptive statistics. Comparisons of two variables with numeric values were performed using *t* test presented as the mean (SD) and Pearson’s χ^2^ test or Fisher’s exact test for categorical values, expressed as the count (percentage) for children in this study (Table 1). 

#### 4.6.2. Telomere Length Measurements

To assess the measurement reliability, the intra-assay %CVs (i.e., the degree of variation of replicates within a qPCR assay) were calculated for the quadruplicates, and inter-assay %CVs (i.e., the degree of variation between assay-to-assay) were calculated for the standards, which were repeated on each plate [107,108]. The intra- and inter-assay ICC that estimates the percent of variance of participants were also calculated as another indicator of measurement reliability [83,109]. Individual ICCs were calculated using a one-way random-effects model of measurements of the same type (i.e., samples, standards, DNA controls, and dilution concentrations), measured in sets of quadruplicates, within and between 48 plates using the Stata command ‘icc’ [110]. Distributions of relative TL at both time points were non-normal based on their respective plots and Shapiro–Wilk tests (Figure 2) (Table 1). TL data underwent logarithmic, and square root transformations and was further evaluated by performing a likelihood ratio test to compare models with and without a transformation. All likelihood ratio tests yielded *p* > 0.05; thus, the original relative continuous non-transformed scale of TL measurements was used in further statistical analyses. The untransformed TL has also been reported to be more biologically relevant [111]. As TL was not normally distributed, Spearman correlation analysis was used to assess the association between TL0 and TL12 (Figure 2). Regression models with robust standard error estimation were used to account for possible heteroscedasticity. 

#### 4.6.3. Univariable and Multivariable Models

Univariable linear or logistic regression models were used to assess the associations between separate TL at each time point and ADHD symptoms. Mutually adjusted models were assessed to account for longitudinal measures of TL for one ‘distal’ outcome [112]. Directed acyclic graphs were used to guide multivariable models. In brief, key factors were separately assessed for independent associations with TL12 and with ADHD symptoms (Table 3) and then subjected to a detailed examination using modern data-adaptive approaches to assess whether other factors were antecedents, mediators of putative exposure-disease associations, or potential confounders [101,113]. Early life factors with a change in exposure-disease association of 10% or more were assessed with mediation analyses [114]. Causal pathways of early life factors operating through TL were assessed by mediation analysis; we used the R statistical package ‘mediation’ [115,116]. Factors that were considered determinants of ADHD symptoms (i.e., independent of TL) were included in the multivariable model to improve precision [117]. When two factors had a strong Pearson correlation, only one was used as a covariate to minimize overadjustment bias [117]. Factors that fulfilled the criteria of confounders were included in the multivariable model (Supplementary Figure S2). 

#### 4.6.4. Additional Analyses

Furthermore, additional analyses were conducted to evaluate the consistency in our findings. For ADHD symptoms, a dichotomized T score equal to or greater than 60 (T60) that indicated the borderline clinical range for both AP and ADHP outcomes were evaluated [118] (Supplementary Table S1). Categorical TL was examined for both time points, with a median cut-off point indicating categories of shorter (below median) and longer (median or higher) (Supplementary Table S2). The latter was treated as the reference group, since shorter TL has been shown to be associated with an increased risk of disease and increased neurological morbidity [16,119]. TL data was categorized into quintiles to assess the nature of the dose-response relationship. A three-step ordinal composite of both TL time points was derived by taking the two quintile variables and re-classifying the categories to both short (reference), one short (Q1 to Q4) and one long (Q5), and both long (both Q5) (Supplementary Table S2). Sex-specific analyses were conducted to examine whether there was effect modification by child’s sex in the TL-ADHD regression model [116]. We examined the association between ADHD symptoms and the exclusion of: (i) TL that were considered as outliers for TL0 (n = 7 participants) and for TL12 (n = 1 participant) as previously described [56,120]; and (ii) cord blood samples with potential maternal contamination (n = 43 participants) [90] (Supplementary Table S4). Inverse probability weighting was applied to the main results to assess the likelihood of selection bias [121].

In the tables, and in line with convention, we have refrained from asserting that an effect (difference) was found/not found on the basis of *p* values of 0.05 or less, an arbitrary threshold [122]. Here, testing the dose-response or trend effects between the exposure and outcomes is indicated as *p*-trend [123]. We used statistical software packages Stata 15.0 (StataCorp, College Station, TX, USA) and R version 3.6.2 (R Core Team, 2020).

## 5. Conclusions

In this study, we report on a prospective association between shorter TL at 12 months of age and increased ADHD symptoms in children at age two years. We also found that infant SHS exposure in the early postnatal period may impact the development of ADHD and may potentially influence TL shortening. A diverse range of early life factors, including parental education, child’s sex, and birth-related factors may play an important role in early TL dynamics, while other modifiable risk factors of childhood ADHD, such as parental age, household income, perceived stress, and parental lifestyle factors, together highlight the importance of environmental influences, and not genetic factors alone, in early life and subsequent development of ADHD in childhood. Our findings support and extend beyond work implicating that TL plays a role in the pathogenesis of childhood ADHD [19] and that short TL may serve as a useful proximal biomarker of adverse childhood neurodevelopmental disorders, including ADHD, warranting deeper investigation in future studies. Additionally, larger longitudinal samples with longer follow-up studies are needed to delineate formally diagnosed ADHD to validate our findings and to deepen the understanding of the molecular mechanisms underlying the association between shortened TL in early life and subsequent adverse neurodevelopmental outcomes.

## Figures and Tables

**Table 1:** Participant characteristics in the Barwon Infant Study.

Characteristics		N	n (%) or Mean [SD]
**Sociodemographic**			
Maternal age at birth, years		676	32.1 [4.3]
Paternal age at birth, years		648	34.0 [5.4]
Maternal education level:		674	
	Year 11 or less		32 (4.7)
	Year 12 or equivalent		235 (34.9)
	Bachelor or Postgraduate degree		407 (60.4)
Paternal education level:		660	
	Year 11 or less		55 (8.3)
	Year 12 or equivalent		339 (51.4)
	Bachelor or Postgraduate degree		266 (40.3)
Mean household income, AUD 10,000		661	2.7 [1.2]
**Prenatal**			
Perceived Stress Scale, score		673	18.1 [6.2]
Edinburgh Depression Score:		509	
	Low risk (<10)		441 (86.6)
	Moderate risk (10–12)		46 (9.0)
	High risk (>12)		22 (4.3)
Antidepressant use		676	32 (4.7)
Tobacco smoking		674	77 (11.4)
Maternal SHS exposure		663	82 (12.4)
Recreational drug use ^a^		671	5 (0.7)
**Perinatal**			
Child’s sex, male		676	357 (52.8)
Prematurity < 37 weeks		676	29 (4.3)
Apgar score at 5 min		666	9.0 [0.9]
**Postnatal**			
Paternal tobacco smoking at six months		651	73 (11.2)
Child SHS exposure at one month		644	15 (2.3)
**Telomere length (relative T/S ratio):**			
Birth		518	0.9 [0.5]
12 months		477	0.8 [0.5]
**ADHD symptoms (T score) at two years:**			
Attention Problems (AP)		676	51.9 [3.7]
Attention Deficit/Hyperactivity Problems (ADHP)		676	51.8 [3.6]

^a^ Recreational drug use other than marijuana. ADHD—attention deficit/hyperactivity disorder; AUD—Australian dollars; N—population size; SHS—secondhand smoke; T/S ratio—telomeric genomic DNA/*β*-globin single-copy gene.

**Table 2:** Associations between telomere length in infancy and ADHD symptoms at age two years.

Telomere Length (T/S Ratio)	AP (T Score)				ADHP (T Score)			
	Univariable		Multivariable		Univariable		Multivariable	
	*β* (95% CI)	*p*	*β* (95% CI) *	*p*	*β* (95% CI)	*p*	*β* (95% CI) *	*p*
**Individual models:**								
TL0 only	0.10 (−0.62, 0.81)	0.79	−0.05 (−0.55, 0.46)	0.86	0.38 (−0.38, 1.14)	0.33	**−0.68 (−1.14, −0.22)**	**0.004**
TL12 only	**−0.60 (−1.14, −0.05)**	**0.03**	**−0.69 (−1.29, −0.10)**	**0.02**	**−0.70 (−1.12, −0.27)**	**0.001**	**−0.92 (−1.39, −0.44)**	**<0.0001**
**Mutually adjusted in the same model**:								
TL0	0.14 (−0.68, 0.96)	0.74	−0.26 (−0.82, 0.30)	0.36	0.57 (−0.30, 1.45)	0.20	0.19 (−0.40, 0.79)	0.52
TL12	−0.54 (−1.14, 0.06)	0.08	**−0.66 (−1.32, 0.002)**	**0.05**	**−0.83 (−1.34, −0.33)**	**0.001**	**−1.12 (−1.68, −0.57)**	**<0.0001**

**Bold** indicates estimates, 95% CI and corresponding *p* < 0.05. * Adjusted for process factors of exposure (child’s age at blood collection, time from blood collection to storage, and cell type composition) and of the ADHD outcomes (child’s sex and child’s age at the time of behavioral assessment), determinants of ADHD symptoms (maternal age, household income, prenatal perceived stress, prenatal antidepressant use, prenatal recreational drug use, prematurity, and Apgar score at 5 min) and also adjusted for a potential confounder (infant SHS exposure at one month). AP—Attention Problems; ADHP—Attention Deficit/Hyperactivity Problems; TL0—telomere length at birth; TL12—telomere length at 12 months; T/S ratio—telomeric genomic DNA/*β*-globin single-copy gene.

**Table 3:** Associations between key early life sociodemographic and environmental factors and TL12 or ADHD symptoms at age two years.

		TL12 (T/S Ratio)				AP (T Score)		ADHP (T Score)	
Factors		*β* (95% CI) *	*p*	*β* (95% CI) †	*p*	*β* (95% CI) ^	*p*	*β* (95% CI) ^	*p*
**Sociodemographic**									
Maternal age at birth, years		0.006 (−0.004, 0.02)	0.24	0.007 (−0.003, 0.02)	0.16	**−0.10 (−0.16, −0.03)**	**0.003**	**−0.10 (−0.16, −0.04)**	**0.002**
Paternal age at birth, years		0.003 (−0.004, 0.01)	0.41	0.003 (−0.005, 0.01)	0.48	**−0.07 (−0.12, −0.03)**	**0.002**	**−0.05 (−0.10, −0.01)**	**0.03**
Maternal education:									
	Year 11 or less	Reference		Reference		Reference		Reference	
	Year 12 or equivalent	**−0.09 (−0.17, −0.007)**	**0.03**	**−0.09 (−0.18, −0.004)**	**0.04**	−1.08 (−2.97, 0.80)	0.26	−0.64 (−2.44, 1.16)	0.48
	Bachelor or Postgraduate degree	**−0.04 (−0.09, 0.0007)**	**0.05**	−0.04 (−0.09, 0.007)	0.10	−1.44 (−3.28, 0.40)	0.13	−1.05 (−2.79, 0.70)	0.24
	*p*-trend		*0.51*		*0.99*		*0.07*		*0.07*
Paternal education:									
	Year 11 or less	Reference		Reference		Reference		Reference	
	Year 12 or equivalent	**−0.08 (−0.16, 0.001)**	**0.05**	**−0.09 (−0.18, −0.001)**	**0.05**	−1.19 (−2.64, 0.26)	0.11	**−1.57 (−3.04, −0.10)**	**0.04**
	Bachelor or Postgraduate degree	−0.04 (−0.08, 0.006)	0.09	−0.04 (−0.09, 0.01)	0.15	**−1.46 (−2.91, −0.01)**	**0.05**	**−1.82 (−3.28, −0.36)**	**0.01**
	*p*-trend		*0.34*		*0.75*		***0.04***		***0.04***
Mean household income, AUD 10,000		0.01 (−0.02, 0.04)	0.57	0.02 (−0.02, 0.06)	0.28	**0.32 (0.04, 0.61)**	**0.03**	**0.35 (0.09, 0.61)**	**0.007**
**Prenatal**									
Perceived Stress Scale, score		0.001 (−0.005, 0.007)	0.75	0.004 (−0.003, 0.01)	0.30	**0.07 (0.02, 0.12)**	**0.004**	**0.08 (0.03, 0.13)**	**0.001**
Edinburgh Depression Score:									
	Low risk (<10)	Reference		Reference		Reference		Reference	
	Moderate risk (10–12)	0.02 (−0.16, 0.21)	0.80	−0.01 (−0.20, 0.18)	0.91	0.74 (−0.55, 2.02)	0.26	0.70 (−0.60, 2.00)	0.29
	High risk (>12)	0.03 (−0.20, 0.27)	0.78	0.06 (−0.19, 0.31)	0.62	0.52 (−0.69, 1.73)	0.40	−0.28 (−1.18, 0.62)	0.54
	*p*-trend		*0.71*		*0.71*		*0.18*		*0.65*
Antidepressant use		0.03 (−0.20, 0.25)	0.83	0.06 (−0.19, 0.31)	0.62	**1.82 (0.02, 3.63)**	**0.05**	1.56 (−0.23, 3.35)	0.09
Tobacco smoking		0.06 (−0.07, 0.19)	0.38	0.03 (−0.01, 0.16)	0.62	0.63 (−0.39, 1.65)	0.23	0.67 (−0.32, 1.65)	0.19
Maternal SHS exposure		−0.02 (−0.14, 0.10)	0.71	−0.02 (−0.15, 0.10)	0.70	0.97 (−0.03, 1.97)	0.06	0.93 (−0.04, 1.89)	0.06
Recreational drug use ^a^		−0.05 (−0.39, 0.29)	0.76	−0.10 (−0.38, 0.18)	0.48	−0.32 (−2.51, 1.87)	0.78	**−0.86 (−1.46, −0.26)**	**0.005**
**Perinatal**									
Child’s sex: male		**−0.09 (−0.17, −0.01)**	**0.03**	**−0.1 (−0.19, −0.009)**	**0.03**	0.42 (−0.13, 0.97)	0.14	0.28 (−0.26, 0.82)	0.31
Prematurity < 37 weeks		0.03 (−0.19, 0.25)	0.79	0.17 (−0.13, 0.46)	0.27	**2.05 (0.20, 3.90)**	**0.03**	1.07 (−0.38, 2.52)	0.15
Apgar score at 5 min		−0.03 (−0.08, 0.006)	0.10	**−0.04 (−0.09, −0.002)**	**0.04**	**−0.4 (−0.79, −0.007)**	**0.05**	**−0.57 (−1.02, −0.12)**	**0.01**
**Postnatal**									
Paternal tobacco smoking at six months		−0.05 (−0.19, 0.10)	0.53	−0.05 (−0.21, 0.12)	0.58	0.62 (−0.24, 1.49)	0.16	**0.94 (0.007, 1.88)**	**0.05**
Child SHS exposure at one month		**−0.29 (−0.53, −0.06)**	**0.02**	−0.27 (−0.58, 0.04)	0.08	**3.34 (0.79, 5.90)**	**0.01**	**2.27 (0.43, 4.12)**	**0.02**

*p* values in *italic* indicates *p*-trend; **Bold** indicates estimates, 95% CI and corresponding *p* ≤ 0.05. * Adjusted for child’s sex, child’s age at the time of blood collection, time interval between blood collection and storage and cell type composition. † Additionally adjusted for telomere length at birth in the same model. ^ Adjusted for child’s sex and child’s age at the time of behavioral assessment. ^a^ Recreational drug use other than marijuana. Additional analysis: Associations between infant SHS exposure at one month and AP (*β* = 2.82; 95% CI (0.28, 5.36); *p* = 0.03) and ADHP (*β* = 1.61; 95% CI (-0.42, 3.63); *p* = 0.12), further adjusted for maternal perceived stress, maternal prenatal smoking, and maternal prenatal SHS exposure. AP—Attention Problems; ADHP—Attention Deficit/Hyperactivity Problems; TL12—telomere length at 12 months; SHS—secondhand smoke; T/S ratio—telomeric genomic DNA/*β*-globin single-copy gene.

## Data Availability

Access to BIS data including all data used in this paper can be requested through the BIS Steering Committee by contacting the corresponding author. Requests to access cohort data are considered on scientific and ethical grounds and, if approved, provided under collaborative research agreements. Deidentified cohort data can be provided in Stata or CSV format. Additional project information, including cohort data description and access procedures, is available at the cohort study’s website http://www.barwoninfantstudy.org.au (accessed on 1 March 2022).

## Supplementary Materials

The following supporting information can be downloaded at: https://www.mdpi.com/article/10.3390/ijms23094601/s1.
