# Supplementary material for: Shortened Infant Telomere Length Is Associated with Attention Deficit/Hyperactivity Disorder Symptoms in Children at Age Two Years: A Birth Cohort Study
Source: Int J Mol Sci. 2022 Apr 21;23(9):4601. doi: 10.3390/ijms23094601 (PMC9104809; doi:10.3390/ijms23094601)
Supplement: Supplementary file 1 [file ijms-23-04601-s001.zip › ijms-1647932-supplementary.pdf]

Supplementary Material

Table of Contents

Figure S1. Flowchart for selection of participants in this study

Figure S2. Association between early life factors and ADHD symptoms at age two years, not mediated by telomere length at 12 months

Table S1. Associations between telomere length in infancy and ADHD symptoms within a borderline to clinical range

Table S2. Associations between categorised telomere length in infancy and ADHD symptoms at age two years

Table S3. Extended associations between early life factors, telomere length in infancy, and ADHD symptoms at age two years

Table S4. Sensitivity analyses on the associations between A) telomere length in infancy with outliers removed; and B) telomere length at birth with potential maternal contamination excluded and ADHD symptoms at age two years

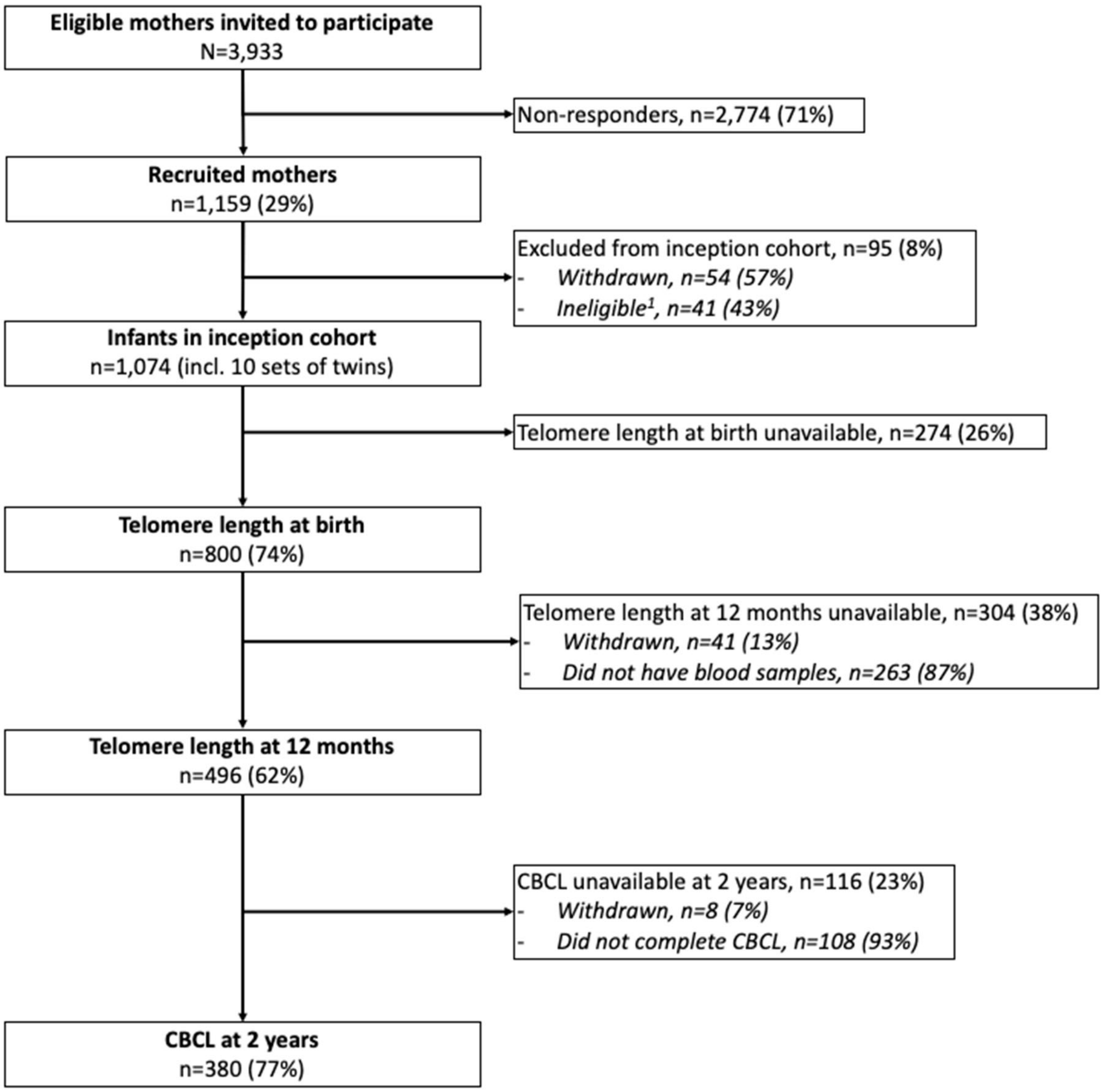

Figure S1. Flowchart for selection of participants in this study

[1] Ineligible include: 8 infants <32 weeks of gestation, 2 cord blood stored privately, 5 infants with major congenital defects, 2 miscarriages, 5 stillbirths, 7 infants with serious illness the first few days and 12 no longer residents. *CBCL Child Behavior Checklist.*

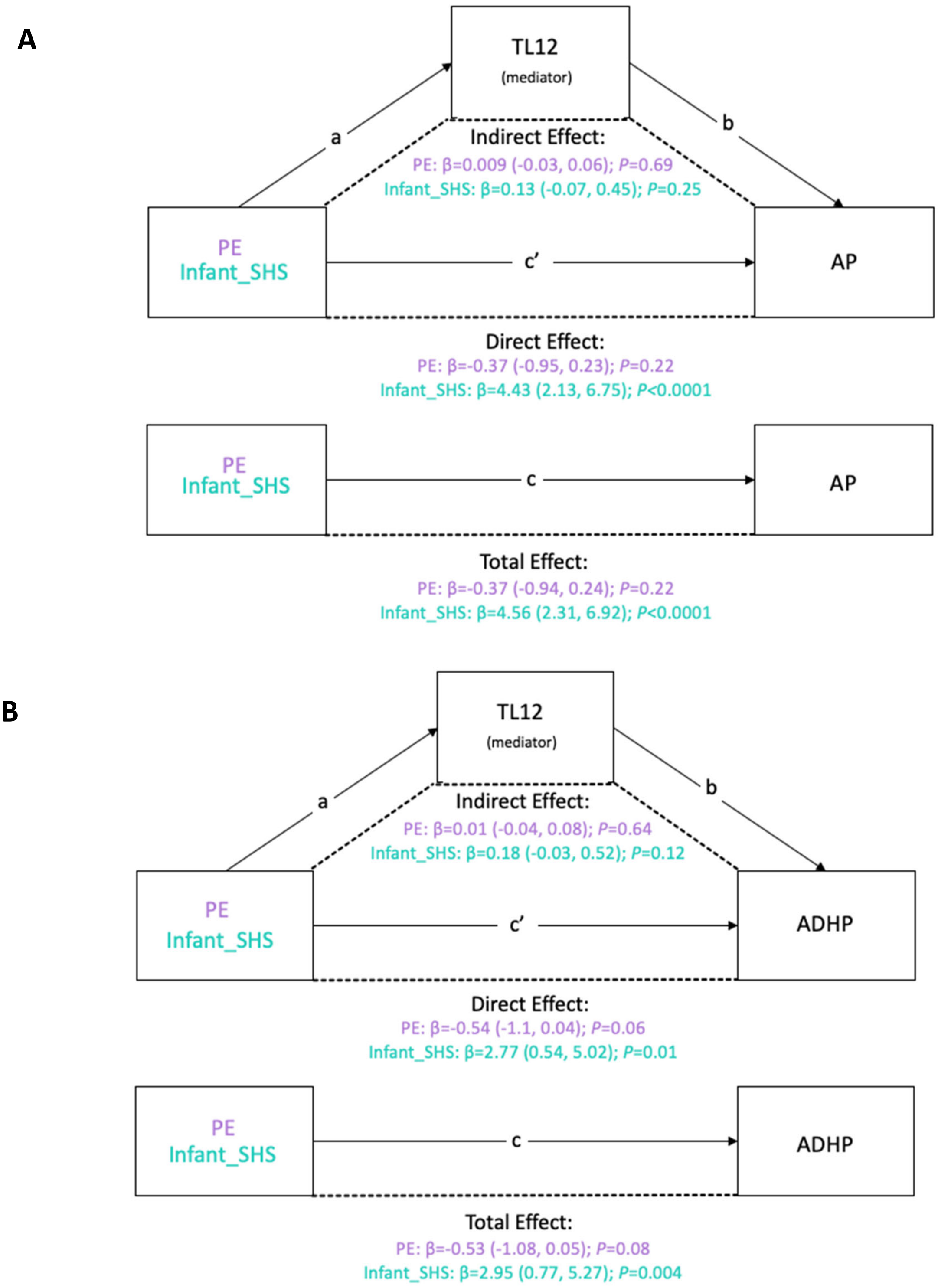

Figure S2. Association between early life factors and ADHD symptoms at age two years, not mediated by telomere length at 12 months

Associations between paternal education (purple) and infant SHS exposure at one month (turquoise) and (A) AP or (B) ADHP at age two years, not mediated by TL12. *AP Attention Problems; ADHP Attention Deficit/Hyperactivity Problems; PE paternal education; SHS secondhand smoke; TL12 telomere length at 12 months.*

Table S1. Associations between telomere length in infancy and ADHD symptoms within a borderline to clinical range

Telomere length (T/S ratio)	AP-T60				ADHP-T60			
	Univariate		Multivariate		Univariate		Multivariate	
	OR (95% CI)	*P*	OR (95% CI) *	*P*	OR (95% CI)	*P*	OR (95% CI) *	*P*
**Individual models:**								
TL0 only	1.33 (0.71, 2.51)	0.37	1.02 (0.47, 2.20)	0.96	1.63 (0.91, 2.92)	0.10	1.55 (0.84, 2.87)	0.16
TL12 only	0.46 (0.20, 1.04)	0.06	**0.26 (0.09, 0.73)**	**0.01**	0.56 (0.27, 1.15)	0.11	**0.39 (0.18, 0.84)**	**0.02**
**Mutually adjusted in the same model:**								
TL0	1.91 (0.89, 4.12)	0.10	1.33 (0.51, 3.51)	0.56	1.99 (0.93, 4.24)	0.08	1.37 (0.53, 3.55)	0.52
TL12	0.49 (0.19, 1.26)	0.14	**0.27 (0.09, 0.82)**	**0.02**	0.49 (0.23, 1.08)	0.08	**0.30 (0.14, 0.65)**	**0.002**

Bold indicates estimates, 95% CI and corresponding *P*<0.05. *Adjusted for process factors of exposure (child's age at blood collection, time from blood collection to storage and cell type composition) and of the ADHD outcomes (child's sex and child's age at the time of behavioral assessment), determinants of ADHD symptoms (maternal age, household income, prenatal perceived stress, prenatal antidepressant use, prenatal recreational drug use, prematurity and Apgar score at 5 minutes) and also adjusted for a potential confounder (infant SHS exposure at one month). *Note.* Small sample size with ADHD symptoms within the borderline to clinical range: AP (n=38); ADHP (n=37). *AP Attention Problems; ADHP Attention Deficit/Hyperactivity Problems; CBCL Child Behavior Checklist; TL0 telomere length at birth; TL12 telomere length at 12 months; OR odds ratio; T/S ratio telomeric genomic DNA/β-globin single-copy gene.*

Table S2. Associations between categorised telomere length in infancy and ADHD symptoms at age two years

		AP (T score)		ADHP (T score)	
**Telomere length (T/S ratio)**		*β* (95% CI)	*P*	*β* (95% CI)	*P*
**Median ^**					
	TL0	-0.09 (-0.75, 0.57)	0.79	0.06 (-0.61, 0.73)	0.86
	TL12	-0.40 (-1.12, 0.31)	0.27	-0.57 (-1.24, 0.10)	0.10
**Quintiles**					
**TL0**	Q5 (longest)	Reference		Reference	
	Q4	-0.11 (-1.15, 0.94)	0.84	-0.46 (-1.57, 0.65)	0.41
	Q3	-0.38 (-1.38, 0.62)	0.46	-0.34 (-1.47, 0.78)	0.55
	Q2	0.42 (-0.76, 1.61)	0.49	-0.18 (-1.38, 1.02)	0.77
	Q1 (shortest)	-0.29 (-1.25, 0.66)	0.55	-0.64 (-1.64, 0.36)	0.21
	P-trend		*0.96*		*0.40*
**TL12**	Q5 (longest)	Reference		Reference	
	Q4	0.07 (-0.79, 0.93)	0.87	0.60 (-0.21, 1.40)	0.15
	Q3	1.41 (0.22, 2.61)	0.02	1.81 (0.66, 2.96)	0.002
	Q2	0.15 (-0.73, 1.03)	0.74	0.51 (-0.30, 1.33)	0.21
	Q1 (shortest)	0.85 (-0.14, 1.84)	0.09	0.91 (0.13, 1.68)	0.02
	P-trend		*0.12*		*0.08*
**Composite**					
	Short-Short	Reference		Reference	
	Short-Long/Long-Short	-0.08 (-0.89, 0.74)	0.86	-0.02 (-0.87, 0.82)	0.96
	Long-Long	**-1.30 (-2.28, -0.32)**	**0.009**	**-1.48 (-2.19, -0.77)**	**<0.0001**
	P-trend		*0.27*		*0.26*
Composite 1 ^		-0.07 (-0.89, 0.74)	0.86	-0.02 (-0.87, 0.83)	0.96
Composite 2 ^		**-1.31 (-2.34, -0.30)**	**0.01**	**-1.56 (-2.33, -0.79)**	**<0.0001**

Bold indicates estimates, 95% CI and corresponding P<0.05; *P* values in *italic* indicates *P-trend*. Adjusted for child's sex, child's age at blood collection and child's age at the time of the behavioral assessment. ^ *Note*: Median cut-off point coded as 0=shorter (below median) vs 1=longer (above median); Composite 1 coded as 0=Short-Short vs 1=One time point short; Composite 2 is coded as 0=Short-Short and 1=Long-Long. *AP Attention Problems; ADHP Attention Deficit/Hyperactivity Problems; CBCL Child Behavior Checklist; TL0 telomere length at birth; TL12 telomere length at 12 months; T/S ratio telomeric genomic DNA /β-globin single-copy gene; Q quintiles.*

Table S3. Associations between extended early life factors, telomere length at 12 months, and ADHD symptoms at age two years

Factors	TL12 (T/S ratio)		AP (T score)		ADHP (T score)	
	β (95% CI) *	*P*	*β* (95% CI) ^	*P*	*β* (95% CI) ^	*P*
**Demographic**						
Maternal ancestry: Caucasian	-0.02 (-0.17, 0.13)	0.82	-0.41 (-1.47, 0.64)	0.44	-1.11 (-2.39, 0.17)	0.09
Paternal ancestry: Caucasian	-0.06 (-0.20, 0.08)	0.38	0.4 (-0.43, 1.23)	0.35	-0.43 (-1.4, 0.54)	0.39
Lone parental status	0.05 (-0.16, 0.26)	0.64	1.11 (-1.21, 3.42)	0.35	1.21 (-0.98, 3.40)	0.28
**Prenatal**						
Pre-conception BMI, kg/m^2	0.0001 (-0.008, 0.009)	0.99	0.03 (-0.02, 0.09)	0.17	0.02 (-0.02, 0.07)	0.36
Family history: Eczema	-0.02 (-0.10, 0.06)	0.65	-0.19 (-0.75, 0.37)	0.51	-0.02 (-0.58, 0.53)	0.93
Preeclampsia	0.19 (-0.04, 0.42)	0.10	0.56 (-1.27, 2.38)	0.55	-0.40 (-1.53, 0.73)	0.49
Gestational hypertension	0.05 (-0.11, 0.20)	0.56	-0.19 (-1.10, 0.72)	0.68	0.32 (-1.04, 1.69)	0.64
Folate levels in RBC (nmol/l)	0.00006 (-0.00008, 0.0002)	0.39	0.0001 (-0.0007, 0.0009)	0.80	0.0003 (-0.001, 0.001)	0.49
Fish oil supplement use	0.05 (-0.10, 0.20)	0.48	-0.42 (-1.2, 0.36)	0.29	-0.55 (-1.25, 0.15)	0.12
Prescription medication use	0.02 (-0.06, 0.11)	0.56	0.23 (-0.34, 0.80)	0.43	-O200.04 (-0.59, 0.52)	0.90
Alcohol use	-0.02 (-0.10, 0.07)	0.68	-0.4 (-0.95, 0.16)	0.16	-0.46 (-1.01, 0.09)	0.10
Smoking during T1	0.06 (-0.07, 0.20)	0.37	0.66 (-0.37, 1.69)	0.21	0.7 (-0.3, 1.69)	0.17
Smoking during T2 and T3	0.13 (-0.15, 0.41)	0.35	0.32 (-1.18, 1.82)	0.67	0.25 (-0.88, 1.38)	0.66
Marijuana use	-0.05 (-0.43, 0.34)	0.81	2.18 (-1.97, 6.32)	0.30	2.38 (-2.36, 7.11)	0.32
**Perinatal**						
Gestational age, weeks	0.02 (-0.009, 0.05)	0.19	-0.18 (-0.43, 0.07)	0.16	-0.07 (-0.29, 0.15)	0.53
Labour duration, hours	-0.005 (-0.01, 0.003)	0.21	0.02 (-0.02, 0.06)	0.38	0.03 (-0.01, 0.07)	0.18
Caesarean section	0.002 (-0.09, 0.09)	0.97	-0.17 (-0.75, 0.41)	0.57	-0.25 (-0.81, 0.31)	0.37
Birth weight z-score [a]	0.008 (-0.04, 0.05)	0.71	0.02 (-0.27, 0.30)	0.91	0.05 (-0.24, 0.33)	0.74
**Postnatal**						
Breastfeeding for ≥6 months	-0.03 (-0.12, 0.05)	0.44	-0.42 (-1.02, 0.18)	0.17	-0.36 (-0.96, 0.23)	0.23
Maternal smoking at 6 months	-0.04 (-0.23, 0.15)	0.67	0.76 (-0.77, 2.30)	0.33	0.67 (-0.53, 1.88)	0.27

Bold indicates estimates, 95% CI and corresponding P<0.05; *P* values in *italic* indicates *P-trend*. * Adjusted for child's sex and child's age at blood collection. ^ Adjusted for child's sex and child's age of behavioral assessment. [a] Birth weight z-score by gestational age and sex according to the United Kingdom WHO child growth standards. *Note*. Associations between extended early life factors and telomere length at birth are being investigated by another colleague. *AP Attention Problems; ADHP Attention Deficit/Hyperactivity Problems; BMI body mass index; RBC red blood cells; TL12 telomere length at 12 months; T1, T2, T3 trimesters 1,2,3; T/S ratio telomeric genomic DNA /β-globin single-copy gene.*

Table S4. Additional analyses on the associations between i) telomere length in infancy with outliers removed; and ii) telomere length at birth with potential maternal contamination excluded and ADHD symptoms at age two years

	AP (T score)				ADHP (T score)			
	Univariate		Multivariate		Univariable		Multivariable	
	β (95% CI)	*P*	*β* (95% CI) *	*P*	*β* (95% CI)	*P*	*β* (95% CI) *	*P*
**i. Telomere length outliers excluded**								
TL0, T/S ratio	-0.02 (-0.66, 0.61)	0.94	0.22 (-0.43, -0.87)	0.51	0.27 (-0.42, 0.97)	0.44	0.46 (-0.21, 1.13)	0.18
TL12, T/S ratio	**-0.71 (-1.23, -0.20)**	**0.007**	**-0.93 (-1.56, -0.30)**	**0.004**	**-0.73 (-1.16, -0.29)**	**0.001**	**-0.93 (-1.46, 0.40)**	**0.001**
**ii. Maternal contamination in cord blood samples excluded**								
TL0, T/S ratio	0.14 (-0.60, 0.87)	0.72	0.08 (-0.50, 0.67)	0.78	0.42 (-0.35, 1.20)	0.29	0.34 (-0.26, 0.94)	0.26

Bold indicates estimates, 95% CI and corresponding *P*<0.05. * Adjusted for process factors of exposure (child's age at blood collection, time from blood collection to storage and cell type composition) and of the ADHD outcomes (child's sex and child's age at the time of behavioral assessment), determinants of ADHD symptoms (maternal age, household income, prenatal perceived stress, prenatal antidepressant use, prenatal recreational drug use, prematurity and Apgar score at 5 minutes) and also adjusted for a potential confounder (infant SHS exposure at one month). *Note.* For i) 7 participants with TL0 and 1 participant with TL12 were outliers; and ii) 43 participants with cord blood samples may potentially have maternal contamination. *AP Attention Problems; ADHP Attention Deficit/Hyperactivity Problems; TL0 telomere length at birth; TL12 telomere length at 12 months; T/S ratio telomeric genomic DNA /β-globin single-copy gene.*
